# Gut microbiota, inflammatory proteins and bone mineral density in different age groups: A Mendelian randomization study

**DOI:** 10.1097/MD.0000000000041875

**Published:** 2025-04-04

**Authors:** Yuechang Hong, Minghui Yang, Xin Xu, Peng Wang, Zixin Ten, Huang Chen, Minqiang Fu, Renying Xiong, Jianjiang Ouyang

**Affiliations:** a School of Clinical Medicine, Jiangxi University of Traditional Chinese Medicine, Nanchang, Jiangxi Province, People’s Republic of China; b Department of Sports Medicine, Affiliated Hospital of Jiangxi University of Traditional Chinese Medicine, Nanchang, People’s Republic of China; c Department of Medicine, Affiliated Hospital of Jiangxi University of Traditional Chinese Medicine, Nanchang, People’s Republic of China.

**Keywords:** bone mineral density, causal relationship, genome-wide association analysis, gut microbiota, inflammatory proteins, Mendelian randomization, sensitivity analysis

## Abstract

Several studies have indicated a potential association between gut microbiota and bone density. However, the causal relationship between gut microbiota and bone mineral density across different age groups, as well as the potential role of inflammatory proteins as mediators, remains unclear. Gut microbiota, inflammatory proteins, and bone mineral density (BMD) were identified in various age groups using summary data from large-scale genome-wide association studies. Mendelian randomization was employed to examine the causal connections between gut microbiota, inflammatory proteins, and BMD in different age groups, primarily utilizing inverse variance weighted as the statistical method. Furthermore, the potential role of inflammatory proteins as mediators in the pathway from gut microbiota to BMD was investigated. Eight positive and 19 negative causal relationships between gut microbiota and BMD were observed across various age groups. We also identified 14 positive and 8 negative causal relationships between inflammatory proteins and BMD in different age groups. Inflammatory proteins did not appear to function as mediators in the pathway from gut microbiota to BMD. Gut microbiota and inflammatory proteins were causally linked to BMD; however, inflammatory proteins did not seem to function as mediators in the pathway from gut microbiota to BMD because the effects of intestinal flora on bone density and the effects of inflammatory factors on bone density were in different directions.

## 1. Introduction

Osteoporosis is a condition characterized by increased bone turnover and decreased bone mass, resulting in bone fragility and an elevated risk of fractures.^[1]^ With the global aging trend, osteoporosis is progressively prevalent and has emerged as a significant public health concern.^[2]^ It is projected that the number of adults with osteoporosis and low bone mass in the United States will reach 71 million by 2030.^[3]^ Bone mineral density (BMD) serves as a crucial diagnostic indicator for osteoporosis, with the World Health Organization defining osteoporosis as a condition where the BMD T-score is <2.5.^[4]^ Anti-resorptive medications are extensively employed in osteoporosis treatment, but they are also associated with substantial limitations and side effects.^[5]^ Recent advances in gut microbiology have unveiled the potential impact of the “bone–gut” axis on the occurrence and progression of osteoporosis,^[6,7]^ offering novel therapeutic avenues for the condition.

The gut microbiota, comprised of bacteria, archaea, fungi, protozoa, and viruses, is now recognized as a crucial factor in regulating host health. It forms a substantial and varied community within the gastrointestinal tract.^[8]^ Stable changes in the gut environment are a risk factor for bone health and a novel potential therapeutic target.^[9]^ The gut microbiota can further influence bone homeostasis through the immune system.^[10]^ The gut microbiota has essential physiological functions linked to nutrition, the immune system, and host defense.^[11]^ Recent research has demonstrated the significant influence of gut microbiota on BMD across different life stages.^[12,13]^ The diversity of gut microbiota linked to BMD seems to display a consistent trend from ages 0 to 45. Furthermore, compared with the 0 to 45 age group, individuals aged 45 and above show a rise in the abundance of BMD-related gut microbiota species, along with a growing prevalence of the Firmicutes phylum by the age of 60.^[14]^

The gut microbiota also plays a crucial role in the regulation of inflammatory proteins,^[15,16]^ and in maintaining a balance between pro-inflammatory and anti-inflammatory responses in the intestinal tract.^[17]^ For instance, inflammatory bowel disease serves as a typical demonstration of the imbalance between the gut microbiome and the immune system within the intestine.^[18]^ Moreover, research has indicated a close association between the gut microbiota and the development of immune-mediated inflammatory diseases such as diabetes, arthritis, and systemic lupus erythematosus.^[19]^

On one hand, the gut microbiota can regulate the body’s chronic inflammatory response, thus influencing the progression of diseases.^[17,20]^ On the other hand, an elevated systemic immune inflammation index represents a risk factor for osteoporosis.^[21,22]^ Both factors appear capable of affecting BMD. Hence, we posit that inflammatory proteins act as intermediaries between the gut microbiota and BMD. Typically, verifying their causal relationship necessitates randomized controlled trials. However, conducting an randomized controlled trials demands significant manpower and resources, and ethical considerations may sometimes hinder its continuation.

Mendelian randomization (MR) is a method used to evaluate causal inference in epidemiological research. It applies genetic variants strongly associated with the exposure factor as instrumental variables (IVs) to assess the causal relationship between the exposure factor and the outcome.^[23,24]^ This approach can help eliminate the influence of confounding factors to determine the true impact of the exposure factor on the outcome.^[25]^ We conducted an MR study based on data from genome-wide association studies (GWAS), to explore whether inflammatory proteins are potential mediators between gut microbiota and BMD, and to identify specific pathogens.

## 2. Materials and methods

### 2.1. Data sources

The genetic variations of the gut microbiota were derived from the 16S rRNA gene sequencing profiles and genotype data of 18,340 participants from 24 cohorts, which were compiled and analyzed by the MiBioGen consortium. This consortium conducted a large-scale, multi-ancestry, genome-wide meta-analysis of common human genetic variations and their associations with the gut microbiome. In carrying out the mapping of microbial trait loci, the aim was to identify genetic loci influencing the relative abundance of microbial taxa.^[26]^ The experiment identified 131 genera with an average abundance exceeding 1%, including 12 unknown genera. Consequently, this study involved the analysis of 119 genus-level taxonomic groups. The GWAS data for bone density at different age groups (0–15 years, 15–30 years, 30–45 years, 45–60 years, and over 60 age) were obtained from the IEU GWAS database at the University of Bristol (https://gwas.mrcieu.ac.uk/terms/) as outcome variables. The GWAS data for inflammatory proteins were derived from a study by Jing Hua Zhao et al, who conducted whole-genome pQTL mapping for 91 plasma proteins measured with the Olink Inflammation Panel in 14,824 individuals of European descent across 11 cohorts and performed a meta-analysis.^[27]^ Furthermore, the IEU Open GWAS database was utilized, and comprehensive GWAS summary statistics data for each inflammatory protein are available in the EBI GWAS database (encoded as GCST90274758–GCST90274848).

The GWAS summary statistics used in this study all come from publicly available databases. Each original GWAS study obtained ethical approval. Furthermore, individual-level data were not used in this study. Therefore, no new ethical review board approval is required.

### 2.2. Research design

**Figure 1.**
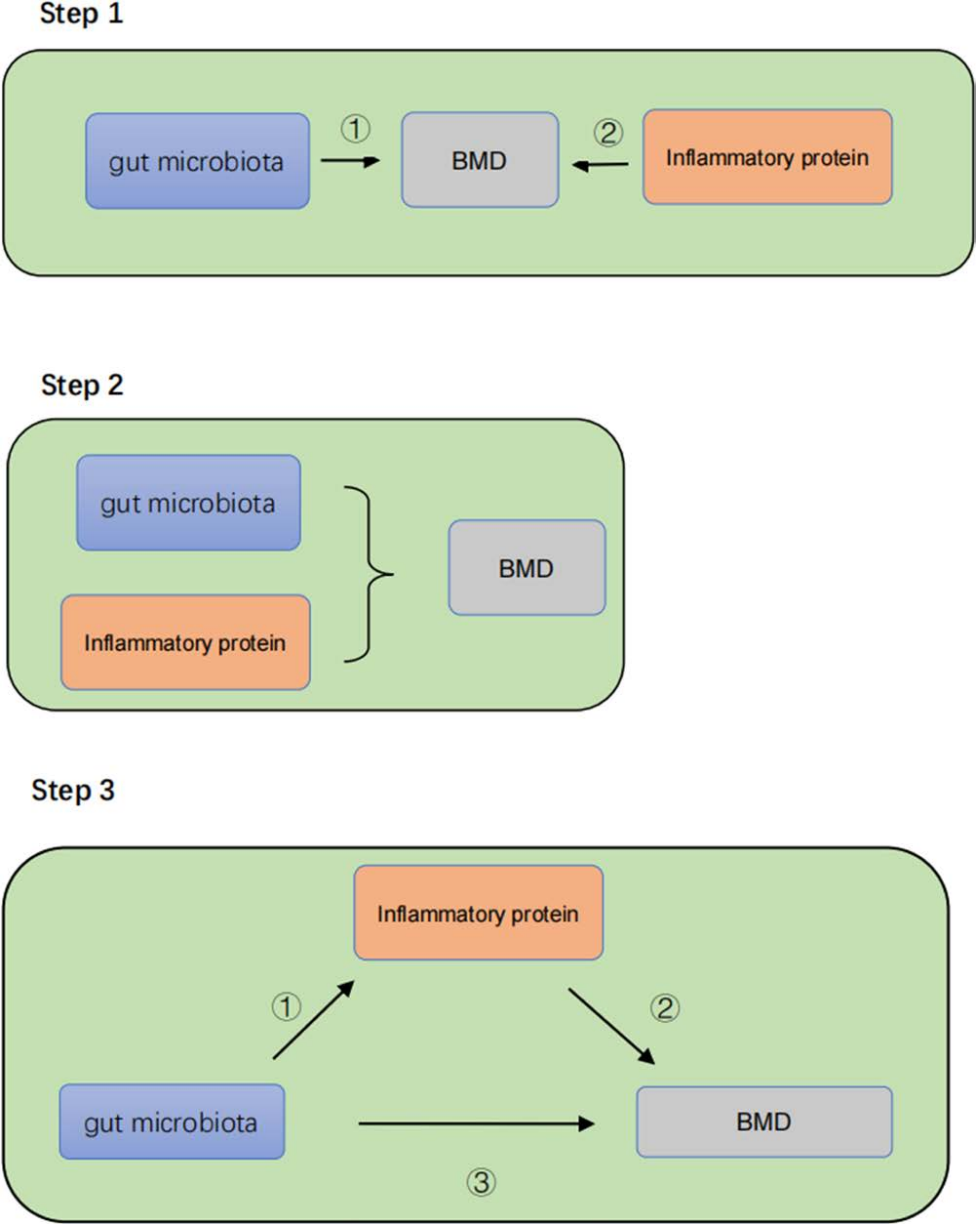
Study overview. Step 1: univariate Mendelian randomization analysis (pathway 1 includes performing a two-sample analysis using gut microbiota as the exposure and BMD as the outcome; pathway 2 includes performing a two-sample analysis using inflammatory protein as the exposure and BMD as the outcome.) Step 2: multivariate Mendelian randomization analysis. Step 3: mediation effect analysis (pathway 1 is the causal effect of gut microbiota on inflammatory proteins; pathway 2 is the causal effect of inflammatory proteins on BMD; pathway 3 is the overall effect of gut microbiota on BMD).

Two-sample MR analyses were performed, utilizing gut microbiota and inflammatory proteins as exposures and different age groups’ BMD as outcomes. Following the identification of positive exposures in the two-sample MR analysis, a multivariable MR analysis was conducted, examining the outcomes. Upon observing consistent directional effects of gut microbiota and inflammatory proteins on BMD in the multivariable MR analysis results, with a *P*-value below .05, the mediation effect of inflammatory proteins between gut microbiota and BMD was explored (Fig. 1).

### 2.3. Screening instrument variables

To ensure the selection of relevant single-nucleotide polymorphism (SNP), those associated with the gut microbiota (*P* < 1.0 × 10^-5^) and inflammatory proteins (*P* < 5.0 × 10^-6^) were identified.^[28]^ Additionally, a minimum F-value of 10 was required (F = beta^2^/se^2^), where beta represents the allele effect value and se indicates the standard error.^[29]^ Subsequently, a threshold of *r*^2^ = 0.001 and a genetic distance of 10,000 kb were set. Any SNPs exceeding *r*^2^ > 0.001 and kb < 10,000 were excluded from the analysis to mitigate potential linkage disequilibrium bias.^[30]^ In cases where a palindromic SNP was present, allele frequency information was utilized to deduce the ancestral allele. Ultimately, the effective SNPs significantly linked with the gut microbiota and inflammatory proteins were obtained as IVs.

### 2.4. MR analysis

#### 2.4.1. Univariate MR analysis

In the investigation, the gut microbiota and inflammatory proteins were utilized as exposures, while bone density in various age cohorts was examined as the outcomes. Two-sample MR analysis was executed, employing the inverse variance weighted (IVW), weighted median method, MR-Egger regression, simple mode, and weighted mode to elucidate causal relationships, with IVW being the primary MR analysis method.^[31–33]^ The IVW combines the MR effect estimates of individual SNPs to derive an overarching weighted estimate of the potential causal effect. It is considered the most reliable when there is no horizontal pleiotropy of IVs. Even when 50% of the information originates from genetic variations of ineffective IVs, the weighted median method can still yield a consistent estimate of the causal effect.^[34]^ Statistical significance is attributed to results when the IVW’s *P*-value is <.05 and it aligns directionally with MR-Egger. Furthermore, MR-Egger, simple mode, and weighted mode analyses were conducted as adjuncts to the IVW method to bolster the robustness of the study results.^[35]^

To assess the causal impact of gut microbiota and inflammatory proteins on bone density, we performed MR analyses using 2 distinct sample sets. Statistical significance was established when the *P*-value of the IVW method was below .05, and the effect’s direction aligned between IVW and MR-Egger. The bilateral *P*-value, following false discovery rate adjustment, indicated statistical significance, set at .0005 (0.05/91) for inflammatory proteins.^[36]^ *P*-values below .05, but above the false discovery rate-corrected threshold, hinted at a potential association.^[28]^

#### 2.4.2. Multivariate MR analysis

Multivariable MR (MVMR) is a method that can decompose the total effect between exposure and outcome into direct and indirect effects through intermediaries,^[37,38]^ allowing for the estimation of causal effects among 3 variables: exposure, mediator, and outcome. MVMR leverages genetic variations associated with multiple potentially correlated exposures to assess the impact of each exposure on an individual outcome, as well as to estimate mediating effects. By harnessing the advantages of causal inference using genetic tools, MVMR can mitigate biases resulting from confounding while facilitating the estimation of diverse effects essential for mediation analysis. Through two-sample MR analysis, this study will incorporate gut microbiota and inflammatory proteins that exert a significant causal effect on BMD into a mediation analysis. Additionally, we will examine gut microbiota that influence relevant inflammatory proteins, conducting a multivariable MVMR analysis with BMD as the outcome variable to further investigate whether inflammatory proteins act as mediators in the pathway from gut microbiota to BMD.

### 2.5. Version and name of statistics software

All MR analyses were done using R (version 4.2.3) and TwoSampleMR and MR-PRESSO packages.

## 3. Results and analysis

### 3.1. Univariate MR analysis

According to the standards of the selected IVs, a total of 2559 independent SNPs from 196 gut microbiota were screened (Table S1, Supplemental Digital Content, https://links.lww.com/MD/O647). Subsequently, we detected 1820 SNPs associated with 91 inflammatory proteins at a significance level of *P* < 5 × 10^-6^ (Table S2, Supplemental Digital Content, https://links.lww.com/MD/O648). All F statistics exceeded 10, signifying a successful mitigation of bias from weak IVs.

To discern the effect of individual SNPs on the outcome, a “leave-one-out” analysis was performed for each instrumental SNP.^[39]^ The association between exposure and outcome risk is presented using the odds ratio (OR) and its 95% confidence interval (CI); a *P*-value of ≤ .05 indicates a potential causal relationship.

In the 0 to 15 age group, the application of the IVW method revealed significant associations with BMD for 3 specific microbial communities, class Gammaproteobacteria, family Victivallaceae, and genus Actinomyces (Table S3, Supplemental Digital Content, https://links.lww.com/MD/O649, Table S5, Supplemental Digital Content, https://links.lww.com/MD/O650). Notably, class Gammaproteobacteria [OR = 1.29, 95% CI (1.07, 1.56), *P* = .006] and family Victivallaceae [OR = 1.08, 95% CI (1.007, 1.16), *P* = .0029] emerged as significant protective factors for BMD in this context. Furthermore, 2 inflammation-related proteins, namely CD40L receptor levels [OR = 1.06, 95% CI (1.004, 1.12), *P* = .033] and programmed cell death 1 ligand 1 levels [OR = 1.10, 95% CI (1.006, 1.21), *P* = .035], were also identified as key protective factors for BMD.

In the 15 to 30 age group, there were 3 types of microbial communities associated with BMD. These include the following protective factors: genus Lachnospiraceae UCG008 [OR = 0.77, 95% CI (0.65, 0.92), *P* = .003], genus Phascolarctobacterium [OR = 0.71, 95% CI (0.54, 0.94), *P* = .01], and genus Ruminococcaceae UCG003 [OR = 0.73, 95% CI (0.56, 0.95), *P* = .02]. In terms of inflammatory proteins, T-cell surface glycoprotein CD5 levels [OR = 0.82, 95% CI (0.71, 0.95), *P* = .008], and urokinase-type plasminogen activator levels [OR = 0.85, 95% CI (0.75, 0.98), *P* = .02] were risk factors for BMD, while matrix metalloproteinase-1 levels [OR = 1.17, 95% CI (1.01, 1.36), *P* = .02] are protective factors.

In the 30 to 45 age group, there were 4 types of microbial communities related to BMD, specifically, genus Eisenbergiella [OR = 0.88, 95% CI (0.80, 0.98), *P* = .02], genus Lachnospiraceae UCG010 [OR = 0.86, 95% CI (0.74, 0.99), *P* = .04], genus Parasutterella [OR = 0.87, 95% CI (0.77, 0.99), *P* = .04], and genus Turicibacter [OR = 0.80, 95% CI (0.70, 0.91), *P* = .001], which were all risk factors for BMD. Regarding inflammatory proteins, beta-nerve growth factor levels [OR = 1.14, 95% CI (1.004, 1.305), *P* = .04], interleukin-17A levels [OR = 1.16, 95% CI (1.01, 1.32), *P* = .02], and interleukin-6 levels [OR = 1.22, 95% CI (1.07, 1.39), *P* = .002] were protective factors, while fibroblast growth factor 21 levels [OR = 0.86, 95% CI (0.78, 0.96), *P* = .009], and leukemia inhibitory factor levels [OR = 0.83, 95% CI (0.70, 0.98), *P* = .03] were risk factors.

In the age range of 45 to 60, 13 types of microbiota were associated with BMD. The family Bifidobacteriaceae (OR = 0.79, 95% CI: 0.70–0.90, *P* = .002), genus Actinomyces (OR = 0.88, 95% CI: 0.80–0.97, *P* = .01), genus Lachnospiraceae UCG004 (OR = 0.84, 95% CI: 0.75–0.93, *P* = .002), and order Bifidobacteriales (OR = 0.79, 95% CI: 0.70–0.90, *P* = .0002) were identified as significant risk factors for BMD. Conversely, the genus Clostridium innocuum group (OR = 1.08, 95% CI: 1.006–1.16, *P* = .01), genus Ruminococcaceae UCG002 (OR = 1.14, 95% CI: 1.02–1.27, *P* = .01), and genus Ruminococcaceae UCG009 (OR = 1.09, 95% CI: 1.004–1.18, *P* = .03) were identified as protective factors for BMD. Eight types of inflammatory proteins were associated with BMD. Axin-1 levels (OR = 1.21, 95% CI: 1.07–1.36, *P* = .001), fibroblast growth factor 19 levels (OR = 1.13, 95% CI: 1.03–1.24, *P* = .005), interleukin-17A levels (OR = 1.13, 95% CI: 1.03–1.24, *P* = .009), and TNF-related activation-induced cytokine levels (OR = 1.07, 95% CI: 1.005–1.15, *P* = .03) were identified as significant protective factors for BMD. Conversely, natural killer cell receptor 2B4 levels (OR = 0.91, 95% CI: 0.86–0.97, *P* = .005) and fibroblast growth factor 21 levels (OR = 0.87, 95% CI: 0.78–0.97, *P* = .01) were identified as risk factors for BMD.

In the over 60 age group, 4 types of microbiota were linked to BMD. The family Peptostreptococcaceae (OR = 1.11, 95% CI: 1.02–1.21, *P* = .01) and genus Lachnospiraceae UCG004 (OR = 1.11, 95% CI: 1.008–1.23, *P* = .03) were identified as protective factors for BMD, while the genus Alistipes (OR = 0.85, 95% CI: 0.77–0.95, *P* = .004) and genus Ruminococcus gauvreauii group (OR = 0.87, 95% CI: 0.79–0.97, *P* = .01) were identified as risk factors for BMD. Additionally, 4 types of inflammatory proteins were associated with BMD, where C–C motif chemokine 4 levels (OR = 1.04, 95% CI: 1.008–1.09, *P* = .01) and TNF-related activation-induced cytokine levels (OR = 1.07, 95% CI: 1.01–1.13, *P* = .01) were identified as protective factors for BMD, and C–X–C motif chemokine 5 levels (OR = 0.94, 95% CI: 0.90–0.98, *P* = .01) and fibroblast growth factor 5 levels (OR = 0.96, 95% CI: 0.93–0.99, *P* = .03) were identified as risk factors for BMD.

### 3.2. Sensitivity analysis

Sensitivity analysis encompasses tests for horizontal pleiotropy and heterogeneity. The presence of horizontal pleiotropy in a study is indicated if a significant intercept term was detected in the MR-Egger regression analysis.^[40]^ Heterogeneity of SNPs can be evaluated through the Cochran *Q* test,^[41]^ with a statistically significant Cochran *Q* statistic (*P* < .05) signifying substantial heterogeneity in the analysis results. Furthermore, the MR pleiotropy residual sum and outlier test (MR-PRESSO) was utilized to identify and remove outliers, followed by the reanalysis of the remaining IVs.^[42]^

**Figure 2.**
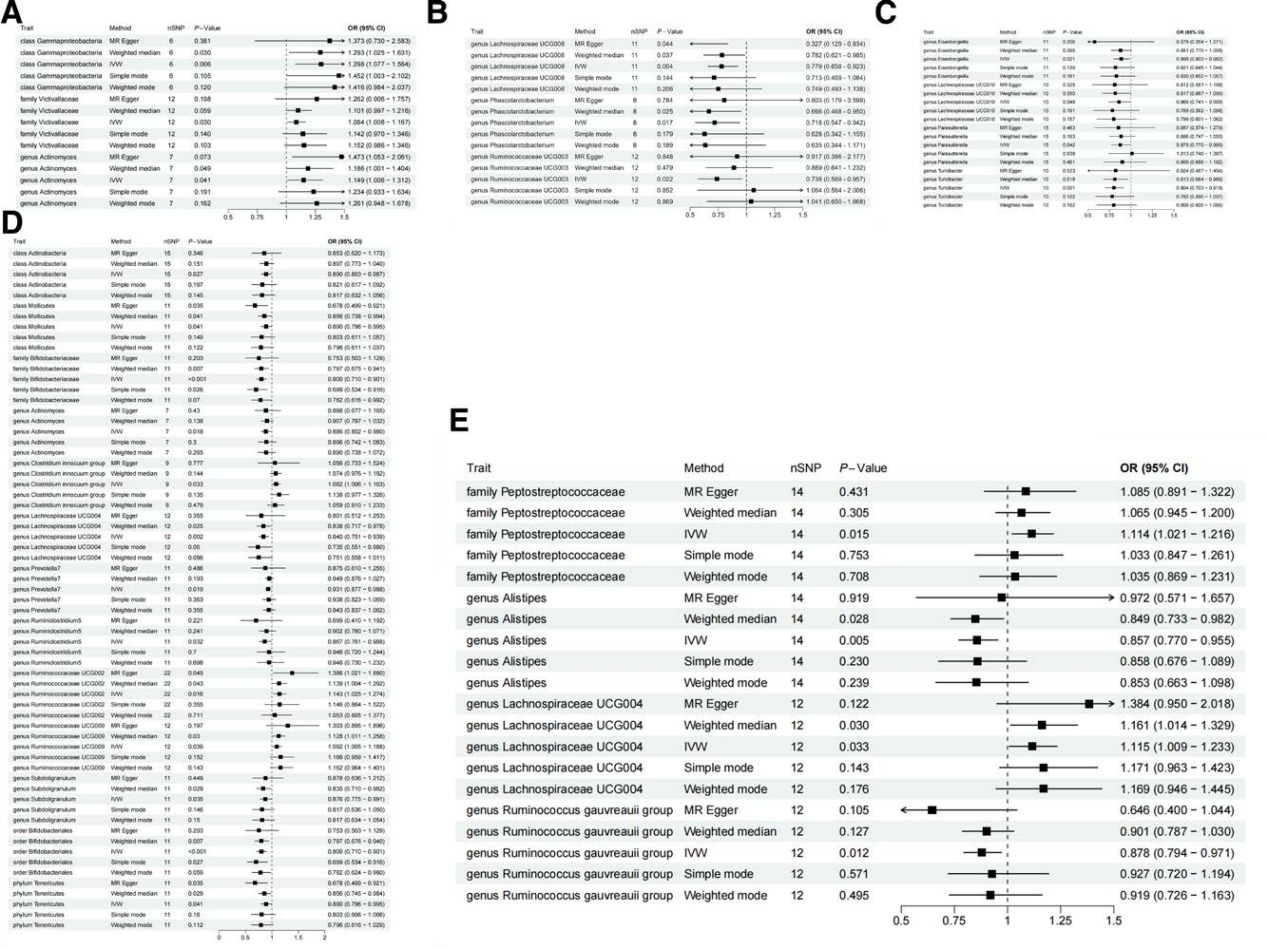
(A) Forest plots for the effect of gut microbiota on BMD (0–15 years). (B) Forest plots for the effect of gut microbiota on BMD (15–30 years). (C) Forest plots for the effect of Gut microbiota on BMD (30–45 years). (D) Forest plots for the effect of gut microbiota on BMD (45–60 years). (E) Forest plots for the effect of Gut microbiota on BMD (>60 years).

**Figure 3.**
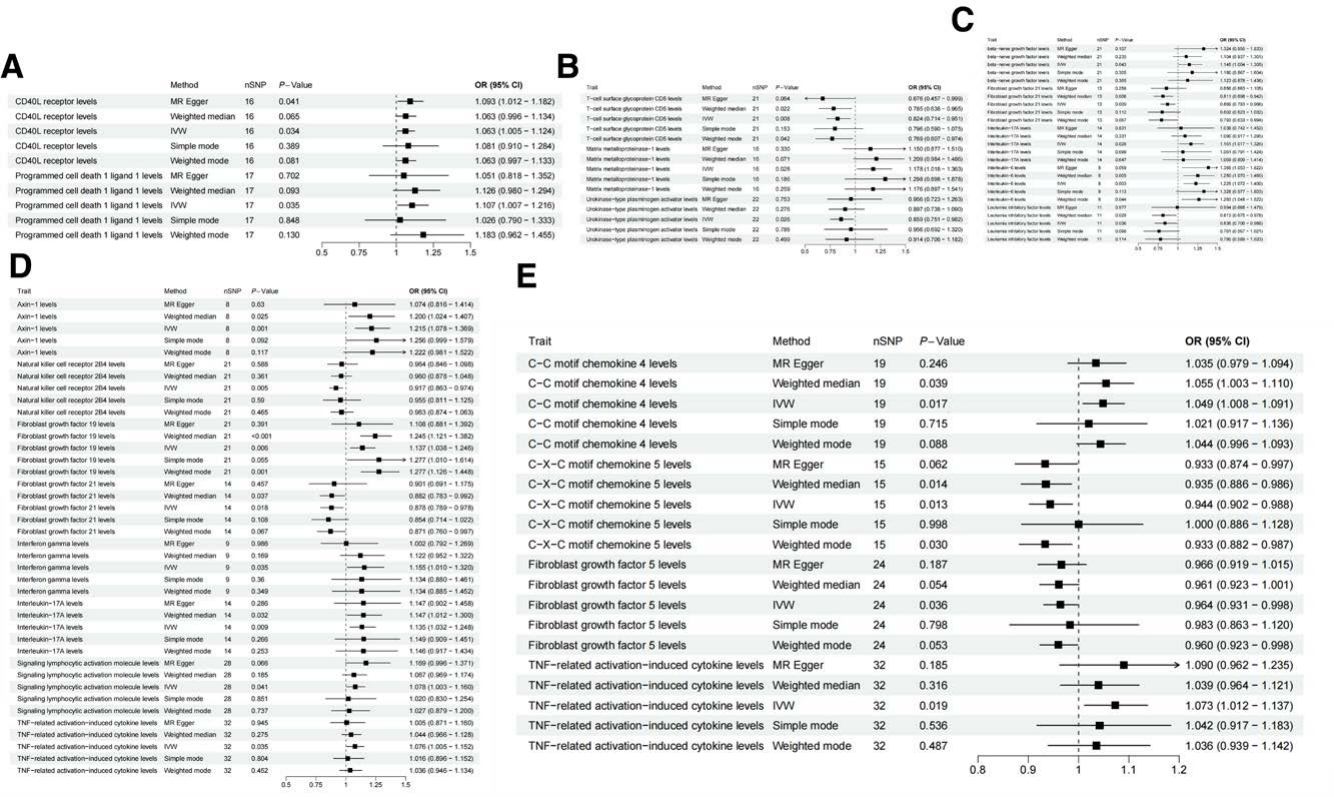
(A) Forest plots for the effect of inflammatory protein on BMD (0–15 years). (B) Forest plots for the effect of inflammatory protein on BMD (15–30 years). (C) Forest plots for the effect of inflammatory protein on BMD (30–45 years). (D) Forest plots for the effect of inflammatory protein on BMD (45–60 years). (E) Forest plots for the effect of inflammatory protein on BMD (>60 years).

Through MR-Egger regression, we evaluated the potential horizontal pleiotropy between SNPs and the results. The *P*-values of the MR-Egger regression intercept were all above .05, suggesting an absence of apparent horizontal pleiotropy. Furthermore, both Cochran test for IVW and MR-Egger indicated Q_pval higher than 0.05, indicating no heterogeneity (Table S4, Supplemental Digital Content, https://links.lww.com/MD/O651, Table S6, Supplemental Digital Content, https://links.lww.com/MD/O652). MR-PRESSO analysis revealed that the levels of the genus Ruminococcaceae UCG002 and fibroblast growth factor 19 had *P*-values lower than .05 for the 45 to 60 age group BMD, with no identified outliers. However, for fibroblast growth factor 21 levels and TNF-related activation-induced cytokine levels, MR-PRESSO analysis identified 1 outlier for each in the 45 to 60 and > 60 age groups, respectively. Following the removal of these outliers, the MR analysis showed no significant associations with BMD. Furthermore, the forest plot illustrated the causal relationship between gut microbiota, inflammatory proteins, and bone density (Figs. 2 and 3).

### 3.3. Multivariate MR analysis

By performing a multivariable MR analysis using the positive exposures identified from the two-sample MR analysis (Table S7, Supplemental Digital Content, https://links.lww.com/MD/O653), we discovered a shared influence on BMD from a group of exposures among the age groups of 30 to 45 and over 60. In the 45 to 60 age group, 5 exposures collectively influence the occurrence of BMD.

### 3.4. Mediation analysis

This study identified a causal relationship between gut microbiota, inflammatory proteins, and bone density. The two-sample MR analysis revealed that in the age groups of 30 to 45 and over 60, a particular type of inflammatory protein may serve as a mediator in bone density, with 5 types of inflammatory proteins potentially acting as mediators in the 45 to 60 age group. In addition, the MVMR analysis indicated that the beta values of all potential mediator inflammatory proteins for bone density show different effect directions from the beta values of gut microbiota for bone density.

## 4. Discussion

To the best of our knowledge, our study is the first to utilize MR to investigate the relationship between gut microbiota and the underlying mechanisms in BMD using inflammatory proteins. Our results demonstrate a causal link between gut microbiota associated with BMD and inflammatory proteins associated with BMD. However, we found that the inflammatory proteins do not act as mediators in the pathway between gut microbiota and BMD. Additionally, differential BMD may be either a cause or a collateral effect of gut microbiota. Therefore, our study is fundamental in addressing gaps in our comprehension of BMD, shedding light on the causative or tangential involvement of gut microbiota and relevant interactions with inflammatory proteins in a genomic context. Thus, investigation into the gut microbiota may offer new therapeutic approaches for osteoporosis.

In this study, we employed MR analysis to explore the potential causal relationship between gut microbiota and BMD among various age groups. We conducted an examination of the abundance of 196 prevalent gut microbiota species and their association with 5 separate age groups. The findings uncovered that specific gut microbiota species function as both risk and protective factors for BMD across diverse age groups.

In the 0 to 15 age group, an increase in the abundance of the class Gammaproteobacteria, family Victivallaceae, and genus Actinomyces was regarded as a protective factor for BMD. Actinomyces was considered a normal bacterium in the digestive tract and has the capacity to produce short-chain fatty acids (SCFAs) and various antibiotics.^[43]^ SCFAs were absorbed in the intestines and then transported to other organs, where they modulated various physiological functions through mechanisms including histone deacetylase inhibition, G protein-coupled receptor signaling, and integration of acetyl-CoA production and metabolism. These combined mechanisms enabled SCFAs to enhance immunity and suppress inflammatory responses in the intestines and other organs.^[44]^ Inflammation also significantly contributed to the pathogenesis of osteoporosis.^[45]^

In the 15 to 30 age group, an elevated abundance of the genus Lachnospiraceae UCG008 was identified as a significant risk factor for BMD. High levels of Lachnospiraceae were positively associated with glucose or lipid metabolism and can contribute to inflammation.^[46]^ An increase in the abundance of the genera Phascolarctobacterium and Ruminococcaceae UCG003 was considered a risk factor for BMD in this age bracket.

In the 30 to 45 age group, the genus Turicibacter played a substantial role in influencing changes in BMD, probably linked to the ability of Turicibacter strains to modify host bile acid and lipid metabolism.^[47]^ Recent epidemiological studies have highlighted a significant association between blood lipid abnormalities and osteoporosis.^[48,49]^ The genera Eisenbergiella, Lachnospiraceae UCG010, and Parasutterella were also identified as protective factors for BMD in this age group.

In the 45 to 60 age group, an increase in the abundance of the family Bifidobacteriaceae, genus Lachnospiraceae UCG004, and order Bifidobacteriales was significantly negatively correlated with BMD. However, a study conducted in Wuhan, China found that individuals with low bone density showed increased BMD with higher levels of Bifidobacterium species. Furthermore, BMD was observed to increase with an increase in Bifidobacterium abundance, This suggests a complex relationship, as a decreased abundance of the family Lachnospiraceae was positively correlated with BMD and T-scores among individuals with low bone density.^[50]^ Additionally, in this age group, the genera Ruminococcaceae UCG002 and Ruminococcaceae UCG009 also showed a positive correlation with BMD, which supports the conclusion that different bacterial populations may influence bone density in varying ways.

In the age group over 60, the genera Alistipes and *Ruminococcus gauvreauii* group exhibited a significant negative correlation with BMD. Alistipes has the ability to ferment protein in the gastrointestinal tract, leading to the production of harmful metabolites such as ammonia, H2S, cresol, indole, and phenol.^[51]^ However, the specific mechanism of its relationship with BMD is still unclear, and there is currently a lack of research on their relationship with BMD. Meanwhile, the family Peptostreptococcaceae and genus Lachnospiraceae UCG004 showed a positive correlation with BMD. The specific impact of genus Lachnospiraceae UCG004 is yet to be fully explored, and its effect contradicts that in the previous age group.

This study examined the impact of gut microbiota on BMD across various age groups. Although the specific mechanism by which gut microbiota influences changes in BMD remains unclear, we postulate that inflammatory factors may act as intermediaries between gut microbiota and BMD. Utilizing MR analysis, we identified 19 inflammatory factors that exhibited correlations with BMD. Specifically, T-cell surface glycoprotein CD5 levels and interleukin-6 levels showed significant correlations with BMD in the 15 to 30 age group, whereas Axin-1 levels, natural killer cell receptor 2B4 levels, and fibroblast growth factor 19 levels were notably associated with BMD in the 30 to 45 age group. Furthermore, interleukin-17A levels and TNF-related activation-induced cytokine levels displayed correlations with BMD across various age groups. Previous studies have illustrated that these pro-inflammatory cytokines stimulate osteoclast bone resorption, impacting BMD.^[45,52]^

This study utilized MR to reduce the influence of confounding factors, thereby enhancing result accuracy significantly. Nevertheless, our research presents several limitations. To begin with, the sampling in our study lacked gender stratification. Given estrogen’s notable impact on bone density, especially postmenopausal women, we could not investigate how gut microbiota affects bone density across genders. Additionally, our analysis was limited to the European population, cautioning against generalizing the relationship between gut microbiota and osteoporosis risk to other ethnic groups. Lastly, even though we examined the mediating effect of inflammatory factors between different levels of gut microbiota and BMD in diverse age groups, further investigation is indispensable to comprehend how gut microbiota influences BMD, considering that inflammatory factors may not always act as mediators.

## 5. Conclusion

This study thoroughly examined the causal associations among gut microbiota, inflammatory factors, and BMD across various age groups. Our analysis revealed 8 affirmative and 19 negative causal connections between gut microbiota and BMD across different age groups. Additionally, we uncovered 14 positive and 8 negative causal relationships between inflammatory factors and BMD in different age groups. There is evidence to suggest that inflammatory factors do not act as intermediaries in the pathway from gut microbiota to BMD.

## Acknowledgments

The authors extend their heartfelt gratitude to the IEU Open GWAS Project and MiBioGen consortium for making the crucial genetic data accessible. We appreciate the collaborative spirit of the scientific community in promoting open data sharing.

## Author contributions

**Conceptualization:** Yuechang Hong.

**Data curation:** Yuechang Hong, Minghui Yang, Minqiang Fu.

**Formal analysis:** Minghui Yang.

**Funding acquisition:** Xin Xu, Zixin Ten.

**Investigation:** Minghui Yang, Huang Chen, Minqiang Fu.

**Methodology:** Xin Xu, Zixin Ten, Huang Chen, Renying Xiong.

**Project administration:** Xin Xu, Peng Wang.

**Resources:** Peng Wang, Huang Chen.

**Software:** Peng Wang, Zixin Ten, Minqiang Fu, Renying Xiong.

**Supervision:** Zixin Ten.

**Validation:** Renying Xiong.

**Writing – original draft:** Yuechang Hong.

**Writing – review & editing:** Jianjiang Ouyang.

## Supplementary Material


